# Time to detection of anemia and its predictors among children living with HIV at Debre Tabor and University of Gondar Compressive Specialized Hospitals, 2020: a multicentre retrospective follow-up study

**DOI:** 10.1186/s12887-021-02616-0

**Published:** 2021-03-30

**Authors:** Ermias Sisay Chanie, Dejen Getanh Feleke, Sintayehu Asnakew, Fisha Alebel GebreEyesus, Aragaw Tesfaw, Wubet Alebachew Bayih, Agimasie Tigabu, Yared Asmare Anyalem, Abraham Tsedalu Amare, Demeke Mesfin Belay, Fentaw Teshome Dagnaw, Biruk Beletew Abate

**Affiliations:** 1 Department of Pediatrics and Child Health Nursing, College of Health Sciences, Debre Tabor University, Debre Tabor, Ethiopia; 2 Department of Psychiatry, school of medicine, College of Health Sciences, Debre Tabor University, Debre Tabor, Ethiopia; 3 Department of Nursing, College of Health Sciences, Wolkitie University, Wolkitie, Ethiopia; 4 Department of Public health, College of Health Sciences, Debre Tabor University, Debre Tabor, Ethiopia; 5 Department of Maternal and Neonatal Health Nursing College of Health Science, Debre Tabor University, Debre Tabor, Ethiopia; 6 Department of Adult health Nursing, College of Health Sciences, Debre Tabor University, Debre Tabor, Ethiopia; 7 Department of Pediatrics and Child Health Nursing, College of Health Sciences, Debre Birhan University, Debre Birhan, Ethiopia; 8 Department of Nursing, College of Health Sciences, Woldia University, Woldia, Ethiopia

**Keywords:** Time to detection, Anemia, Predictors, Children, ART, Ethiopia

## Abstract

**Background:**

Even though antiretroviral therapy access for HIV infected children increased dramatically, anemia have been continued as a challenge regardless of a cluster of differentiation (CD4) count and viral load. Hence, this study aimed to assess the time to detection of anemia and its predictors among children living with HIV at Debre Tabor and university of Gondar compressive specialized hospital, 2020.

**Methods:**

A retrospective follow-up study was conducted from January 2010 to December 2018. A total of 372 children under the age of 15 who had received ART were included in the study. Data were collected from children’s medical charts and ART registration logbook using a standard checklist. Besides, the data were entered into Epi data 4.2.2 and then exported to Stata 14.0 for further analysis. The Cox regression model, the variables having *P*-value ≤.05 with 95% CIs in multivariable analysis were declared as statistically significant for anemia.

**Result:**

The mean (±SD) of follow-up periods were 56.6 ± 1.7 SD months. The overall median survival time free from anemia was 137 months, and the incidence rate of anemia was 6.9 per 100 PYO (95% CI: 5.3, 7.8). Moreover, WHO clinical staging of III/IV [AHR: 4.2, 95% CI: 1.80, 11.1], low CD4 count below threshold [AHR: 1.9, 95% CI: 1.09, 3.37], cotrimoxazole preventive therapy non-users, and poor level of adherence [(AHR: 2.4, 95% CI: 1.20, 4.85] were the main predictors of the time to detection of anemia.

**Conclusion:**

The incidence rate of anemia in our retrospective cohort was high. The risk of anemia is present in children living with HIV infection but the risk for anemia is increased based on (WHO clinical staging III and IV, CD4 count below the threshold level, CPT non-users, and poor level of adherence). Since many of these risk factors are present routinely, even within one single patient, our clinical monitoring for anemia quarterly was fully justified as was our routine switch from standard therapies such as AZT to another regimen upon lab confirmation of anemia. Additional methods to improve cotrimoxazole preventative therapy and level of adherence are also needed.

## Background

Hematological abnormalities are a common and independent poor prognostic marker of HIV disease [1, 2], which worsens quality of life [3], and decreases survival [4]. Moreover, hematological abnormalities, mainly anemia has been continued as a challenge in resource limited setting [5].

Anemia is the commonest disorder that is seen among HIV infected children [6]. WHO estimates that over 2 billion people are anemic worldwide, of these, more than 100 million anemic children were living in Africa [7]. Even though ART access for HIV infected children increased dramatically, side effects have continued after the initiation of therapy predominantly in resource-limited setting [3]. Besides, anemia has been a strong risk factor for disease progression and subsequent death regardless of a cluster of differentiation (CD4) count and viral load [4, 8].

Hematological abnormalities have been documented as the second cause of morbidity and mortality in HIV-infected children living in resource-limited countries [9]. Indeed, anemia is common [10]. Anemia is the leading hematologic complication of HIV infection occurring in approximately 30% of patients with an asymptomatic infection and as many as 75–80% of those with Acquired immunodeficiency disease (AIDS) [11], particularly in children whose immune systems are not yet fully developed [12]. Additionally, children are the most vulnerable population for anemia, it is responsible for a high proportion of HIV-related deaths each year [7], up to 90% of children develop anemia during HIV infection [13].

Anemia is a leading cause of hospital admission, reduced cognitive development, growth, and poor immune function in children [7]. since the etiology and pathogenesis of anemia are complex [14] and may be caused by a multitude of factors such as cancer, micronutrient deficiencies; infections, such as malaria and tuberculosis (TB) [15]. In HIV-infected children, the virus can infect parts of the bone marrow responsible for manufacturing red blood cells and erythropoietin, which is required to stimulate red blood cell production [16]. In addition, the antiretroviral medication causes and exacerbates anemia due to bone marrow suppression [17].

Giving attention to the young age of children [18] and children with unemployment parent [19], have been risk factors identified in the past with an impact on HIV associated anemia. Moreover, children with viral load > 1000 copies/ml [9, 20], low CD4 count <200cells/ul [21, 22], and WHO stage III/IV) [7, 20, 23], have been risk factors reported in the previous study.

Despite the above prevention and management approach that has been implemented in Ethiopia, the burden of anemia in HIV infected children is still unacceptably high. Anemia has attracted the attention of both government and researchers since there has been no prior study on time to detection of anemia and its predictors among HIV infected children in the country in general and in the study area in particular. Hence, this study aims to assess the time to detection of anemia and its predictors among children living with HIV at Debre Tabor and university of Gondar compressive specialized hospitals.

## Methods

### Study design and period

A retrospective follow-up study was conducted from January 1, 2010–December 30, 2018, at Debre Tabor and university of Gondar compressive specialized hospitals.

### Study setting

Debre Tabor compressive specialized hospital is the largest hospital in South Gondar Zone which provide in outpatient, inpatient and operation theatre department. The hospital has been provided service for a population of 2.4 million including ART services. The university of Gondar hospital is a 400-bed hospital and the regional referral center for the for four district hospitals in the area. The hospital services about four million across the region^.^

### Source population

All children < 15 years living with HIV at Debre Tabor and university of Gondar comprehensive specialized hospitals.

### Study population

All children < 15 years living with HIV from January 1, 2010–December 30, 2018, at Debre Tabor and university of Gondar Compressive specialized hospitals.

### Inclusion criteria

All children < 15 years living with HIV from January 1, 2010–December 30, 2018, at Debre Tabor and university of Gondar compressive specialized hospitals.

### Exclusion criteria

Medical charts and ART registration logbook of the study participants with incomplete outcome (i.e., hemoglobin status not recorded) variables were excluded.

### Sample size and sampling procedure

The sample size was calculated by using Log-rank survival data analysis of the two-population proportion formula based on the following important assumptions- 95% confidence level, 80% optimum statistical power, and taking type one error 5%. By considering a study was conducted in eastern Ethiopia [24], by taking sex as a predictor variable (on the male as the exposed group denoted by q1 (0.38) and female group denoted by q0 (0.53), and then the total sample size, after adding 10% as incomplete medical records, and the final sample size was 372.

**Fig. 1:**
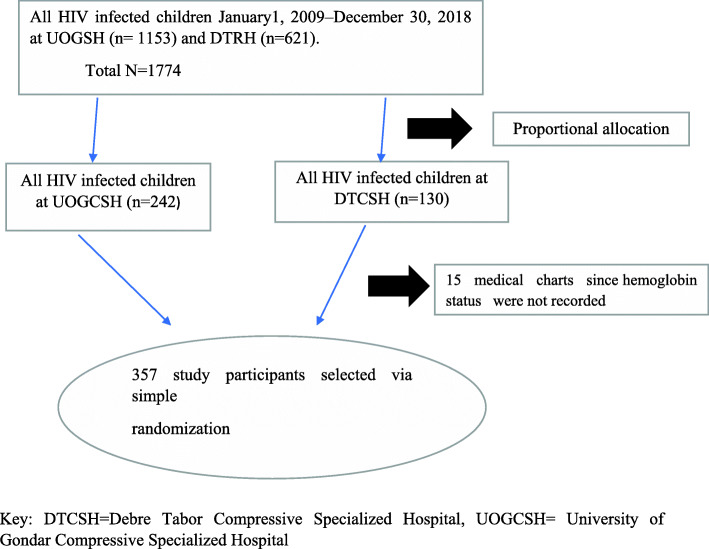
Schematic diagram of sampling procedure predictors among living with HIV at Debre Tabor and university of Gondar compressive specialized hospitals, Northwest, Ethiopia 2020 (*n* = 357)

A total of 372 children who started ART during the study period were identified in the pediatric ART clinics, the investigator assigned the registration numbers from January 1, 2010, to May 30, 2020, in chronological order. Of these, the investigator drew 357 samples that fulfilled the inclusion criteria after reviewing the medical charts and ART registration logbook; and then 15 medical records did not fulfill the inclusion criteria were excluded (Fig. 1).

The dependent variable of this study is time to anemia, whereas age caregivers, sex caregivers, age child, sex child, educational status caregivers, marital status caregivers, occupation caregivers, religion caregivers, residence child, relationship to the child, ART regimen, duration of ART, level of adherence, OI prophylaxis (IP& CPT), developmental milestone/Functional status, treatment failure, history of TB, WHO clinical stage, CD4 count/percent, nutritional status (WGT-Age, WGT-H &HT-Age), and history of OI were independent variables.

### Operational definitions

#### Time to detection of anemia

time calculated by subtracting dates from children ART initiation to the occurrence of the event (i.e., anemia) during the follow-up period.

#### Censored

A study participants who were lost to follow-up, transfer to another service, and end of the study period before developing anemia were considered as censored.

#### Anemia definitions by age

The hemoglobin level less than 11 g/dl for children < 5 years old, < 11.5 g/dl for children 5–11.9 years old, and < 12 g/dl for children 12–14.9 years old [13].

#### Diagnosis of TB

TB is diagnosis by using strong clinical suspicion, laboratory tests, or X-ray examination.

#### General treatment algorithm if anemia occurred

The hemoglobin status of children livening with HIV were monitored every 03 months regularly. However, if they have clinical suspicion, their hemoglobin was checked regardless of the time. If the hemoglobin level is low based on the above reference age group, the treatments are given including regimen change like Stavudine replaced zidovudine if a child had a Hb < 8 g/dl, iron supplementation. In addition to this, blood transfusion was also given if the hemoglobin level < 4 mg/dl.

**Underweight or stunting** was defined as weight for age Z-score < − 2 Standard deviation (SD) under-five children and BMI (Body Mass Index) for age Z-score < − 2 SD for older children [25].

**ART adherence**: defined as good, fair, or poor by the percentage of drug dosage calculated from the total monthly doses of ART drugs taking (Good > 95%, fair 85–94%, poor < 85%) [26].

Treatment failure classified as clinical, immunological and virological failure. Clinical failure: indicating advanced or severe immune deficiency (WHO clinical stage 3 and 4 clinical condition with exception of TB) after 6 months of effective treatment, Immunological failure: Persistent (at least 2 CD4 measurements) CD4 levels below 200cells/mm for children younger than 5 years and, CD4 levels below 100 cells/mm for older than 5 years, and Virological failure, the Viral load above 1000 copies/mL based on two consecutive viral load measurements in 3 months [27, 28].

The first-line ART regimen comprised the nucleoside reverse transcriptase inhibitors usually zidovudine (AZT) in the study setting during the study period. However, AZT or another regimen can change by another drug include drug-resistant viral isolates, anemia, or other adverse outcome based on the laboratory’s investigation or the healthcare providers decision in the study setting. Such as, AZT replaced by Stavudine if a child had a hemoglobin < 8 g/dl. Additionally, Efavirenz replaced nevirapine if a child was on concurrent treatment for tuberculosis with rifampicin.

#### Cotrimoxazole preventative therapy (CPT)

CPT is a feasible, inexpensive, and well-tolerated way of using cotrimoxazole intervention in PLWHA to reduce HIV/AIDS-related morbidities and mortalities caused by various bacteria, fungi, and protozoa. Cotrimoxazole suspension contains 200 mg/40 mg per 5 ml of syrup. Single strength tablets contain 400 mg/80 mg, double-strength tablet twice that. It is possible to divide the tablets for children and infants [29, 30].

#### WHO stages I – IV

According to the Ethiopian ART guidelines, the WHO stages I – II OIs includes Asymptomatic infection, Herpes zoster, and Persistent generalized lymphadenopathy. In addition, the WHO stages III-IV OIs includes unintentional weight loss of more than 10% body weight, recurrent upper respiratory tract infections, chronic diarrhea > 1 month, Prolonged fever > 1 month, Oral candidiasis, TB, HIV wasting syndrome, Pneumocystis carinii (juvenii) pneumonia (PCP), Toxoplasmosis of the brain, Extrapulmonary tuberculosis, Kaposi’s sarcoma, and HIV encephalopathy [31–33].

### Data collection tools and procedures

Data extraction tool was derived from a standard national HIV follow-up form of Ethiopia. Data were extracted from medical charts and ART registration logbook in terms of socio-demographic variables of the children and parents/caregivers contained 10 items. Likewise, clinical and treatment-related variables, which contained a total of 12 items were extracted from medical charts and ART registration logbook. The three nurses, experienced in ART clinic practice collected the data for the study and all were duly qualified with university degrees.

### Data quality control

A Pretest was conducted among 19(5%) of the sample size in medical records to check the consistency of the abstraction tools at University of Gondar comprehensive specialized hospital. Two days of training were given about the objectives, significance, and variables of the research for 03 data collectors and 02 supervisors. All data collectors and supervisors were given basic training and mentorship in ART. The filled formats were checked for completeness by the supervisor, data cleaning and double data entry were carried out to check for any inconsistencies, coding errors, missing or out of range values.

### Data processing and analysis

Data were entered into Epi data 4.2.2 then exported to Stata version 14 for further analysis. Categorical data were presented as frequency and continuous data as the median and interquartile range (IQR). The incidence rate of anemia was calculated with children with anemia as the numerator divided by total child-months. The cumulative probability of anemia was estimated using the Kaplan-Meier failure method and the survival curves were compared between the predictor variable by the log-rank test. In Cox regression analysis, variables having a *p*-value ≤0.25 were entered into multivariable and the variables having ≤.05 in multivariable were declared as statistically significant for anemia. Additionally, the model and the variable were tested via graphic and numerical assessment (global test = 0.644).

## Results

### Socio-demographic characteristics

Out of 372 records, 357 pediatric ART records with a completeness response rate of 95.9% were reviewed and 15 of the medical records were excluded from the study. The median age of children and caregivers was 7.3(SD + -0.28) and 34.9(SD + -0.37) years, respectively. More than half the children 184(51.5%) were male, whereas 193(54.1%) caregivers were female. About 285(79.8%) of the caregivers had urban residences and 249(69.7%) were married. The majority of the caregivers 302(84.6%) were orthodox Christian; likewise, 330 were Amara ethnic group. Moreover, 147(41.2%) and 193(54.1%) of the caregivers were uneducated and unemployed, respectively, and 203(56.9%) of the children were enrolled before a test and treat strategies (Table 1).

**Table 1:** Socio demographic characteristics among children living with HIV at Debre Tabor and university of Gondar Compressive Specialized Hospitals, Northwest, Ethiopia 2020 (*n* = 357)

Exposure variable	Responses	Frequency (*n* = 357)	Percent (%)
Age of the Caregiver’s	≤34 years	216	60.5
	> 34 years	141	39.5
Sex Caregiver’s	Male	164	45.9
	Female	193	54.1
Age of the child (years)	≤7	178	49.9
	> 7	179	50.1
Sex	Male	184	51.5
	Female	173	48.5
Residence	Rural	72	20.2
	Urban	285	79.8
Caregiver’s Marital status	Married	249	69.7
	Widowed	37	10.4
	Divorced	71	19.9
Ethnicity	Amhara	338	94.7
	Tigray	17	4.8
	Others	2	0.6
Religion	Orthodox	302	84.6
	Muslim	49	13.7
	Other^a^	6	1.7
Caregiver’s educational status	No education	147	41.2
	Primary education	92	25.8
	Secondary education	96	26.9
	Tertiary education	22	6.2
Caregiver’s Occupation	Non employed	193	54.1
	Employed	164	45.9

Others, Oromo or Afar and Other^a^: protestant or catholic religion

### Clinical and treatment related characteristics

A total of 70(19.6%) and 111(31.1%) of children had CD4 counts below the threshold level and WHO clinical III and IV, respectively. Forty-one (11.5%) had tuberculosis and 181 (50.7%) had a history of opportunistic infection in the follow-up period. The majority 309(86.6%) of children were on a zidovudine-containing regimen contain, and 291(81.5%) had taken Cotrimoxazole preventive therapy. Only 109 (30.5%) had taken isoniazid preventive therapy. Regarding nutrition, 182(59.1%) and 146(40.9%) of the children were underweight and stunted, respectively. Thirty-six (10.15) of the children had treatment failure, and 199(55.7%) of the children were < 60 months on ART (Table 2).

**Table 2:** Clinical and treatment related characteristics among children living with HIV at Debre Tabor and university of Gondar compressive specialized hospitals, Northwest, Ethiopia 2020 (*n* = 357)

Exposure variable	Responses	Frequency (*n* = 357)	Percent (%)
Weight for height	Normal	175	49.0
	Underweight	182	59.1
Height for age	Normal	211	59.1
	Stunted	146	40.9
CD4 counts or %	Below threshold	70	19.6
	Above threshold	287	80.4
WHO stages	Stage I&II	244	68.3
	Stage III&IV	111	31.1
Hx of OIs	Yes	181	50.7
	No	176	49.3
Regimen at baseline	AZT-based	309	86.6
	d4T-based	48	13.4
Cotrimoxazole preventive therapy (CPT)	Yes	291	81.5
	No	66	18.5
Isoniazid preventive therapy (IPT)	Yes	109	30.5
	No	248	69.5
ART adherence	Good	278	77.9
	Fair	31	8.7
Treatment failure	Poor	48	13.4
	Yes	36	10.1
TB status	No	321	89.9
	Yes	41	11.5
ART duration	No	316	88.5
	< =5 years	199	55.7
Follow-up status Anemia	> 5 years	158	44.3
	Yes	58	16.2

*CD4* cluster of differentiation 4, *WHO* World Health Organization, *OIs* opportunistic infections, *AZT* azidothymidine or Zidovudine, and d4T: stavudine

### The median survival time to develop anemia during the follow-up period

**Fig. 2:**
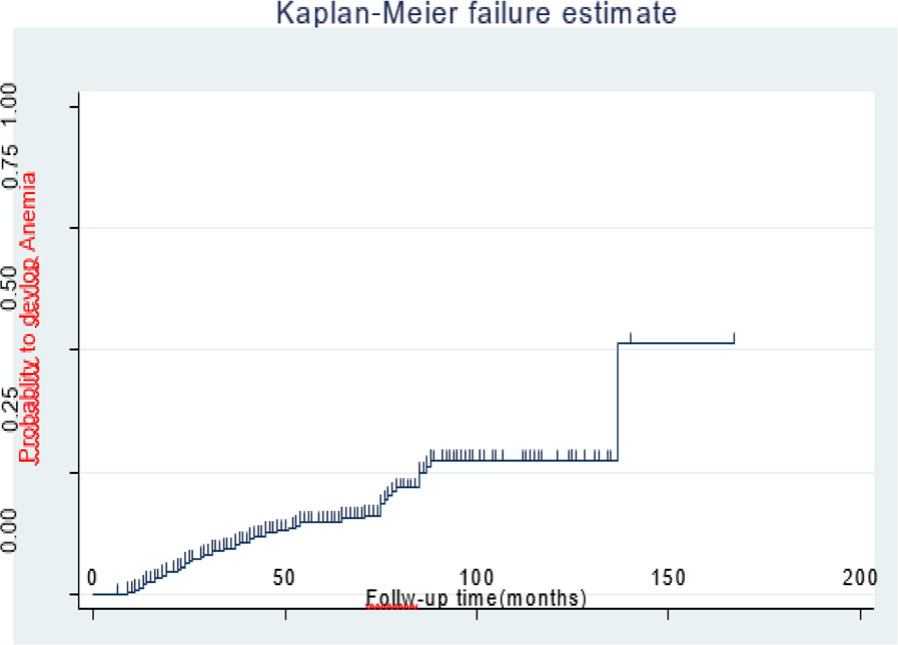
Kaplan-Meier failure estimate of anemia among children living with HIV at Debre Tabor and university of Gondar compressive specialized hospital, Northwest, Ethiopia 2020 (*n* = 357)

**Fig. 3:**
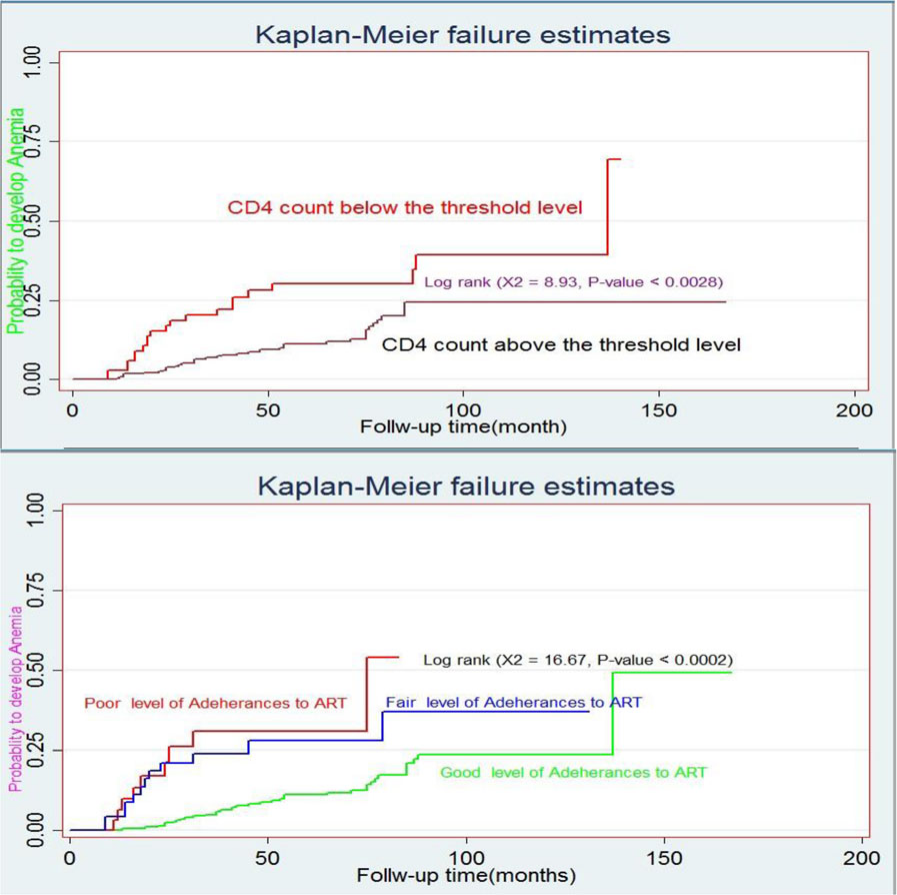
Kaplan-Meier failure estimate of anemia of the main predictors among children living with HIV at Debre Tabor and university of Gondar compressive specialized hospitals, Northwest, Ethiopia 2020 (*n* = 357). Kaplan-Meier failure estimate of anemia of the main predictor variable among children living with HIV at Debre Tabor and university of Gondar compressive specialized hospitals, Northwest, Ethiopia 2020 (*n* = 357)

**Fig. 4:**
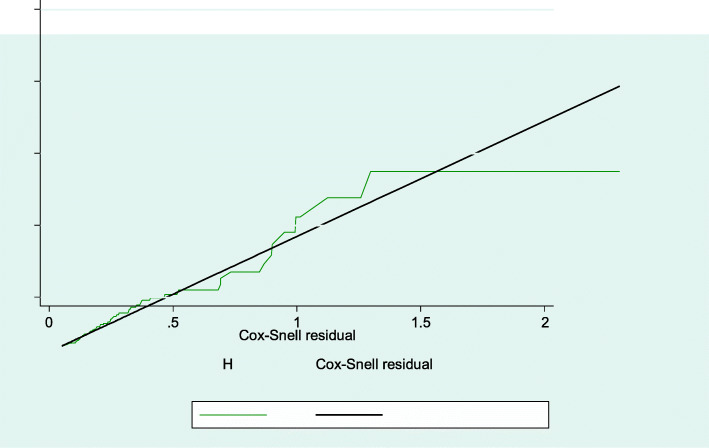
Cox-Snell residual Nelson- Alen cumulative hazard graph for the goodness of model fitness which shows the hazard function follows the 45^o^ closeness among children living with HIV at Debre Tabor and university of Gondar compressive specialized hospital, Northwest, Ethiopia 2020 (*n* = 357)

The mean follow-up periods were 56.6 ± 1.7 SD months, overall yielding 20,191 child-month observations. At the end of follow-up, 58[16.5, 95% CI: 12.8 to 20.4%] of the children were anemia while 299(83.8%) of children were non-anemic. The incidence rate of anemia was 6.9 per 100 PYO (95% CI: 5.3, 7.8). The overall median survival time of children free from anemia was 137 months (Fig. 2). The median survival time free from anemia among children who had a CD4 count below the threshold level was 62 months, but 125 months for those who had a CD4 count above the threshold level. The study revealed, the median survival time free from anemia was 70 months among children who had WHO stage III and IV, but 107 months for those WHO stage I and II in the follow-up period. The median survival time free from anemia among children who had zidovudine regimen were 60 months, while 140 months for those non-zidovudine regimens contained to in the follow-up period (Fig. 3). Log rank survival curve comparisons of the associated predictor variables were estimated. Besides, the Cox-Snell residual nelson- Alen cumulative hazard graph for the goodness of model fitness also evaluated (Fig. 4).

In Cox proportional hazard model bivariable analysis including sex of caregivers, occupation of caregivers, test and treat strategies, weight for age, height for age, TB status, regimen type, CPT, CD4 count threshold level, level of adherence, WHO clinical staging, and OI status were having *P* value less than 0.25 for the occurrence of anemia and entered into the multivariable analysis. Besides, in multivariable models, WHO clinical staging III and IV, CD4 count below the threshold level, CPT non-users, and poor level of adherence were associated with the time to detection of anemia.

The hazard of anemia in children who had WHO stage III and IV were 4.2 times higher as compared to children with WHO staging I and II [AHR: 4.2 (95% CI: 1.80, 11.1)]. Besides, Children who had a CD4 count below the threshold level had an increased hazard of anemia of 1.9 times that of children with a CD4 count above the threshold level [AHR: 1.9 (95% CI: 1.09, 3.37)]. Moreover, children who had cotrimoxazole preventive therapy non-users were 2.2 times higher the hazards of anemia than those who those cotrimoxazole preventive therapy users [AHR: 2.2 (95% CI: 1.23, 3.82)], and children with a poor level of adherence have increased a hazard of anemia by 2.4 times as compared with children with a good level of adherence [(AHR: 2.4 (95% CI: 1.20,4.85)] (Table 3).

**Table 3:** Bivariable and multivariable Cox-regression analysis of predictors for anemia among children living with HIV at Debre Tabor and university of Gondar compressive specialized hospitals, Northwest, Ethiopia 2020 (*n* = 357)

Characteristics		Anemic		HR (95% CI)		
		Yes (58)	No (299)	CHR	AHR	*P*-value
Age of the Caregiver’s	≤34 years	34	182	0.9(0.55–1.57)	–	
	> 34 years	24	117	Ref	–	
Sex Caregiver’s	Male	30	134	1.4(0.83–2.32)	1.3(0.82–2.38)	0.220
	Female	28	165	Ref	Ref	
Age of the child (years)	≤7	25	153	0.8(0.45–1.28)	–	
	> 7	33	146	Ref	–	
Sex	Male	29	155	1.0(0.59–1.66)	–	
	Female	29	144	Ref	–	
Residence	Rural	10	62	0.8 (0.45–1.75)	–	
	Urban	48	237	Ref	–	
Marital status	Married	39	210	0.9(0.49–1.70)	–	
	Divorced	5	32	0.7(0.27–2.09)	–	
	Widowed	14	57	0.8(0.34–2.41)	–	
Religion	Orthodox	49	253	1.4(0.18–10.8)	–	
	Muslim	8	41	1.3(0.16–11.3)	–	
	Other^a^	1	5	Ref	–	
Caregiver’s educational status	No education	25	122	0.7(0.28–1.89)	–	
	Primary education	15	77	0.6(0.23–1.77)	–	
	Secondary education	13	83	0.6(0.20–1.55)	–	
	Tertiary education	5	17	Ref	–	
Occupation of the caregiver	Non employed	37	156	1.6(0.94–2.76)	2.1(0.97–3.89)	0.056
	Employed	21	143	Ref	Ref	
Weight for height	Normal	21	154	Ref	Ref	
	Underweight	37	145	1.9(1.10–3.24)	1.5(0.88–2.72)	0.127
Height for age	Normal	28	183	Ref	Ref	
	Stunted	30	116	1.5(0.92–2.59)	1.2(0.69–2.05)	0.543
CD4 counts or %	Below threshold	21	49	**2.4(1.39–4.09)**	**1.9(1.09–3.37)**	**0.024**
	Above threshold	37	250	Ref	Ref	
WHO stages	Stage I&II	24	220	Ref		
	Stage III&IV	34	77	**4.3(2.50–7.24)**	**4.2(1.8–11.1)**	**0.001**^b^
Hx of opportunistic infections	No	17	159	Ref	Ref	
	Yes	41	140	2.7(1.56–4.85)	1.1(0.45–2.85)	0.800
Regimen at baseline	AZT-based	52	257	1.4(0.60–3.24)	–	
	d4T-based	6	42	Ref	–	
Cotrimoxazole preventive therapy (CPT) Isoniazid preventive therapy (IPT)	No	22	44	**3.1(1.82–5.32)**	**2.2(1.23–3.82)**	**0.008**^b^
	Yes	36	255	Ref	Ref	
	No	40	208	1.0(0.55–1.72)	–	
	Yes	18	91	Ref	–	
ART adherence	Good	39	241	Ref	Ref	
	Fair	9	22	4.3(2.02–8.98)	2.0(0.88–4.79)	0.094
	Poor	12	36	**2.8(1.45–5.37)**	**2.4(1.20–4.85)**	**0.016**^a^
Treatment failure	Yes	5	31	1.0(0.38–2.42)	–	
	No	53	268	Ref	–	
TB status	Yes	11	30	2.6(1.34–5.00)	0.7(0.32–1.53)	0.369
	No	47	269	Ref	Ref	
Duration on ART	<=5 years	43	156	1.2(0–3-3.2)	–	
	> 5 years	15	143	Ref	–	
ART initiation	after test and treat	20	134	Ref	Ref	
	before test and treat (pre-ART)	38	165	1.5(0.89–2.62)	2.0(0.98–3.57)	0.057

^a^Significant at < 0.05; ^b^ Significant at < 0.01; *CHR* Crude hazard ratio, *AHR* adjusted hazard ratio, *Ref* reference category, *CI* confidence interval

## Discussion

This study assessed the time to detection of anemia and its predictors among children living with HIV at Debre Tabor and university of Gondar compressive specialized hospital, Ethiopia. Almost one-in-six HIV infected children (16.5%, *n* = 58) with anemia were recorded in the follow-up period. The incidence rate of anemia was 6.9 per 100 child-years. Moreover, the median survival time was 137 months. WHO clinical staging III/IV, CD4 count below the threshold level, CPT non-user, poor adherence, and before test and treat strategies (pre-ART) were found to be the main predictors of anemia.

The rate of anemia in our study is 16.5% (95% CI: 12.8 to 20.4%), which is comparable with a study conducted in northwest Ethiopia, which were 19.8 and 12.26% [34, 35], and also with Addis Ababa, Ethiopia, which was 17.59% [36]. This could be justified in two ways. Firstly, a cut-off value of Hgb to define anemia. Secondly, study participants had a similar age group (i.e.., children < 15 years). In addition to this, it may be due to the data extraction tools of all studies were prepared from a standard HIV/AIDS Care and treatment guidelines ART service in Ethiopia.

However, The rate of anemia in our study is lower than studies conducted in India, where the incidence was 21.9% [37] and 47.1% [38] respectively., in Jimma, Ethiopia, was 21.9% [37], in Nigeria among aged between 5 and 12 years, was 54.2% [39], in Cameron, was 49.6% [12], in South Africa, was 73% [40], in Gahan 2091 among under 5 Years, was 53.8% [41], Malawi, was 45.7% [9], in Mozambique among 6–59 months of children, was 88% [7], in Kenya, was 35.9% [23], and in Tanzania, was 27.7% respectively [19]. This difference could be due to the cut-off point of anemia, study design since most of the above studies were cross sectional, whereas our study was a cohort. In addition, the variations might be due to the difference in the study area, duration of time on ART, and differences in the follow-up periods. A shorter follow-up period is likely to find a higher probability of anemia when compared with a study with a longer follow-up period. Besides, the discrepancy may be due to monitoring of anemia every 3 months in our study area whereas more frequently in other settings. Likewise, the discrepancy may be due to the study participants (i.e., aged b/n 5–12 years and under 5 years).

Moreover, the finding of this study lower is lower than the study conducted among non-HIV negative children with some malaria endemic areas in Ethiopia [42–44].

This discrepancy can be explained by malaria is a leading cause of anemia due to causes haemolysis of infected, uninfected erythrocytes rapidly [45, 46], but the study setting is not malaria endemic areas. On the other hand, the incidence rate of anemia has been observed in this study is higher than a study conducted in Hawassa Ethiopia, in 2018, which was 11.4% [47]. In the Asia-Pacific region, between January 2003 and September 2013, severe anemia was 5.5% [48], a study conducted from November 2007 to June 2009 in the region of Africa, 4.8% of children with severe anemia [20] in 2016 Uganda, Anemia was 11.8% among 12–14 years respectively [49]. This difference could be explained by the differences in the outcome measurement, ethnicity, sample size, study design, and follow-up period. Moreover, the heterogeneity of the data inherent in a multinational cohort, the type’s health care service, and study setting.

Children who were WHO clinical staging III and IV increased the hazard of anemia by 4.2 times as compared to children with WHO clinical staging I and II. The median duration of free from anemia was significantly shorter in persons with WHO clinical staging III and IV in the follow-up period. The finding is in line with a study from Mozambique, west Africa, and Kenya [7, 20, 23]. This may be due to having advanced WHO clinical staging which could compromise immunity and can lead to severe illness due to viral replication, depletion of CD4 count, and the added burden of disease. Moreover, children who had a CD4 count below the threshold level were increased the hazard of anemia by 1.9 times as compared to children with a CD4 count above the threshold level. The median survival time free from anemia was significantly longer in persons with a CD4 count above the threshold level. This finding was also supported by several studies conducted in Asia, Malawi, and Nigeria [3, 9, 39]. Moreover, the finding was also similar in a study conducted in Black Lion and Zewuidtu referral hospital in Ethiopia < 350 cells/μl [21, 22]. This could be explained by low CD4 count elicited dysfunction of the immune system and increased vulnerability of the host to infection, immunological deficiency that enhances the severity of the disease, and delayed recovery time and increase viral load across time.

Children who were CPT non-users increased the hazards of anemia 2.2 times than those who had those CPT users. A study conducted in Ethiopia revealed that CPT non-users anemia is an independent predictor of anemia [13]. Moreover, the median survival time of free from anemia was significantly shorter in persons with CPT non-users. Indeed, CPT can prevent or reduce the occurrence of opportunistic infection and further complication, therefore, it is important to increase the immune status of the children to decrease viral replication which increases their survival rate by preventing and treating OI infection of which is supported, by a study conducted northwest Ethiopia [50]. CPT prophylaxis has been recommended for the benefit of HIV/AIDS-infected individuals to prevent opportunistic infection since it is a simple and effective intervention to reduce morbidity and to improve quality of life [51]. Besides, children presenting with a poor level of adherence have increased the hazard of anemia by 2.4 times as compared to children with a good level of adherence. Furthermore, the median survival time to develop anemia was significantly longer in persons with a good level of adherence. This could be explained the poor level of adherence has been shown to influence the natural history of HIV disease by accelerating the rate of disease progression, opportunistic infection, and mortality. Moreover, counselling about the importance of adherence has been provided in ART service to prevent opportunistic infection since it is a simple and effective intervention.

This study has some limitations. First, data were collected from routine medical care records and there were limited data on possible predictors of anemia, such as viral load level and psychological support. Second, age, educational, and occupation status of the care givers, as the presence of these variables might be causal for the occurrence of anemia during the initiation of ART.

## Conclusion

The incidence rate of anemia in our retrospective cohort was high. The risk of anemia is present in children living with HIV infection but the risk for anemia is increased based on (WHO clinical staging III and IV, CD4 count below the threshold level, CPT non-users, and poor level of adherence). Since many of these risk factors are present routinely, even within one single patient, our clinical monitoring for anemia quarterly was fully justified as was our routine switch from standard therapies such as AZT to other regimens upon lab confirmation of anemia. Additional methods to improve cotrimoxazole preventative therapy and level of adherence are also needed.

## Data Availability

Data will be had upon request from the corresponding author.
